# Supplementary figures and images for: Novel Plasmodium falciparum metabolic network reconstruction identifies shifts associated with clinical antimalarial resistance
Source: BMC Genomics. 2017 Jul 19;18:543. doi: 10.1186/s12864-017-3905-1 (PMC5518114; doi:10.1186/s12864-017-3905-1)

A

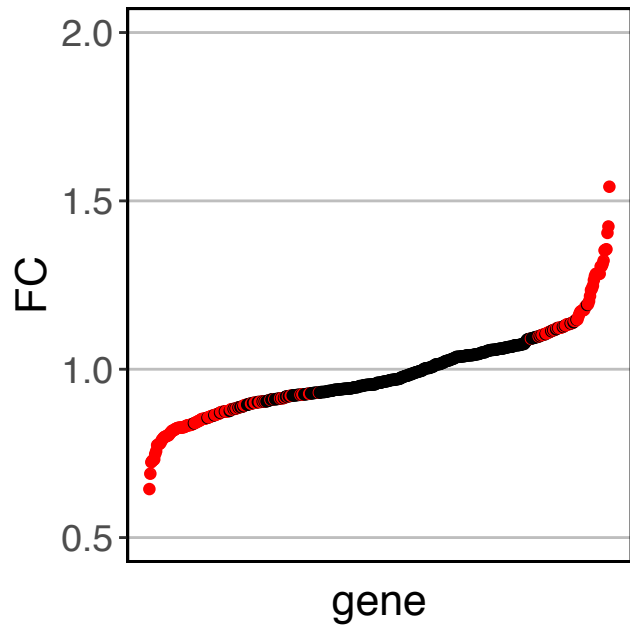

B

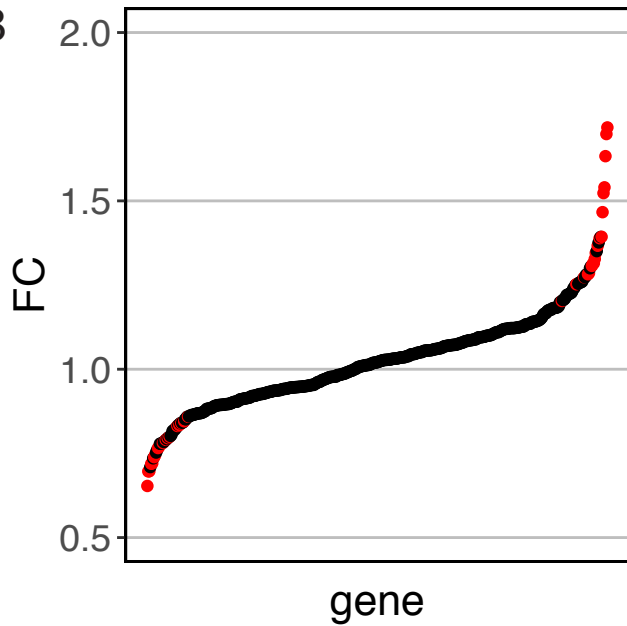

Supplement: Supplementary file 1 — Distribution of genome-wide expression data demonstrates moderate differential expression between sensitive and resistant parasites. Fold change values from differential expression between sensitive and resistant parasites from Cambodia (A) and Vietnam (B) with significantly differentially expressed genes in red. Fold change is the ratio of mean expression in resistant parasites to mean expression in sensitive parasites, for each respective country. (PDF 820 kb) [file 12864_2017_3905_MOESM1_ESM.pdf]

A

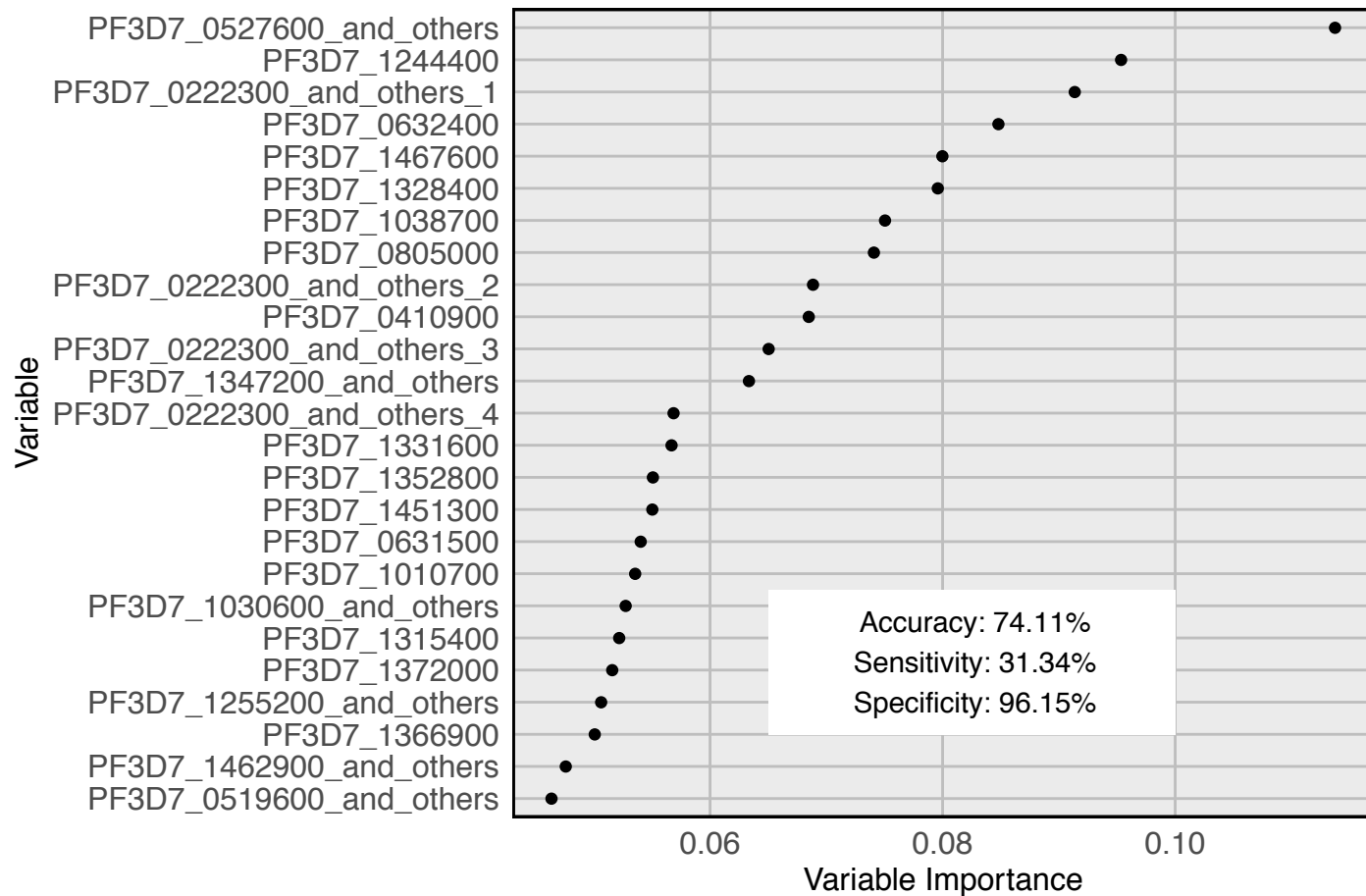

B

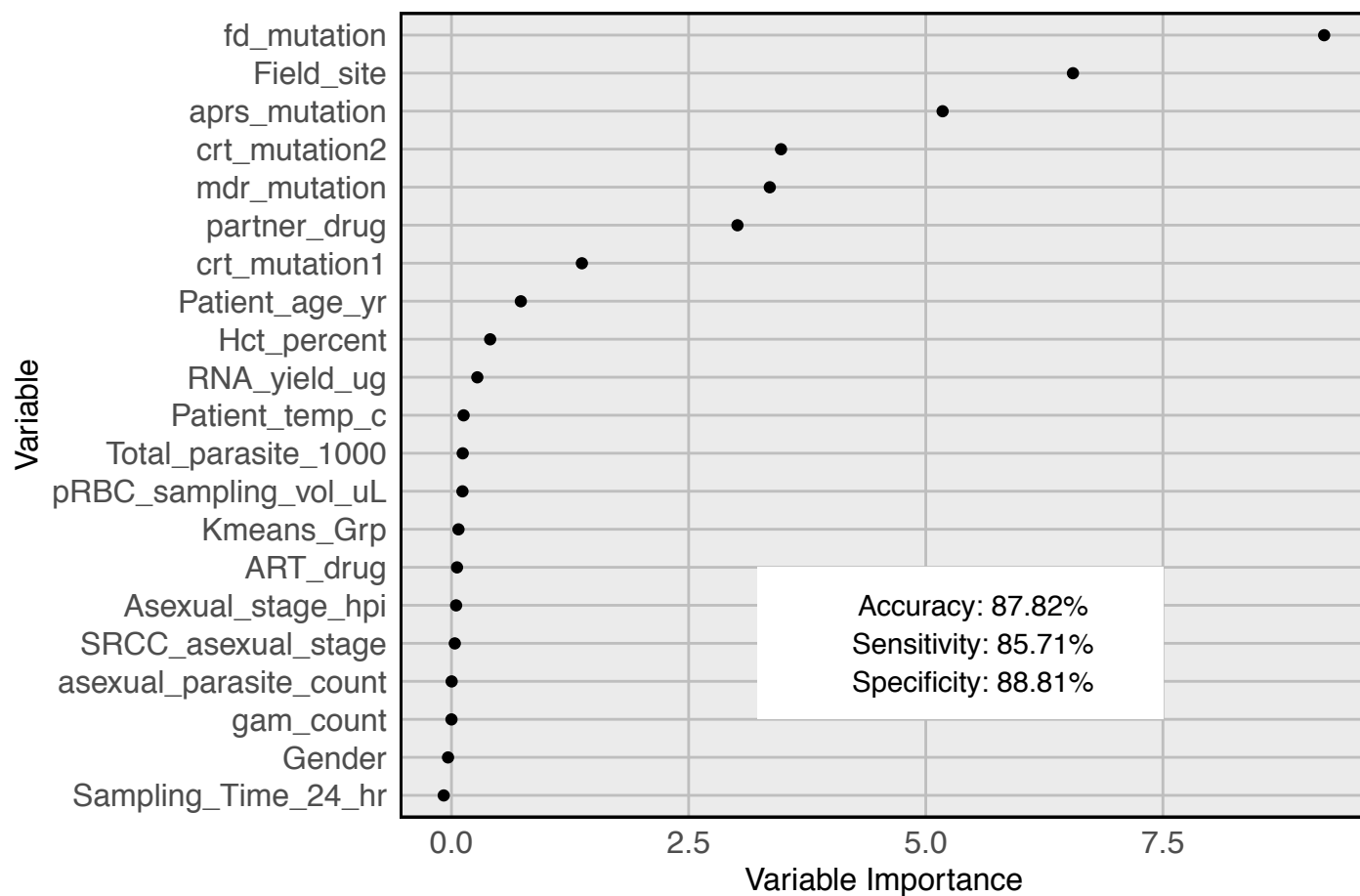

Supplement: Supplementary file 2 — Artemisinin resistance is better predicted by metadata classifier than expression classifier. Using full expression profile (A) or metadata (B), including patient and parasite features (see Methods), we can classify samples as artemisinin sensitive or resistant by Random forest analysis. Of the top 25 most important variables (gene probes) in the expression classifier, 12 encoded exported proteins, four genes of complete unknown function, three encoded a putative kinase and putative phosphatases, one encoded a component of dynein, four were uncharacterized genes though to be involved in protein folding or trafficking, and one encoded a transcription factor. Abbreviation key: (all from [49] unless noted otherwise). aprs_mutation = apicoplast ribosomal protein S10 (PF3D7_1460900.1) mutation [14]; fd_mutation = ferredoxin (PF3D7_1318100) mutation [14]; Field_site = location at which blood sample was collected; mdr_mutation = multidrug resistance protein 2 (PF3D7_1447900) mutation; partner_drug = Partner drug (Artemisinin based combination treatment) administered from day 3 onwards; crt_mutation2 = second CRT (PF3D7_0709000) [14] mutation measured; crt_mutation1 = first CRT (PF3D7_0709000) [14] mutation measured; Patient_age_yr = patient age in years; pRBC_sampling_vol_uL = Volume of packed RBC collected (uL); RNA_yield_ug = Amount of Total RNA isolated for each sample (μg); Patient_temp_c = patient temperature at time of admission in Celsius; ART_drug = Type and dosage of artemisinin drug given once a day on days 0, 1 and 2; asexual_parasite_count = Total asexual parasite densities per uL on admission; total_parasite_1000 = total number of parasites in whole sample of infected RBC collected (pRBC collection vol. * total parasite count per uL) divided by 1000; SRCC_asexual_stage = Spearman rank correlation coefficient of the gene expression for the isolate sample to the projected hpi; Kmeans_Grp = expression group (see [49]); Asexual_stage_hpi = Projected hours post inv [file 12864_2017_3905_MOESM2_ESM.pdf]

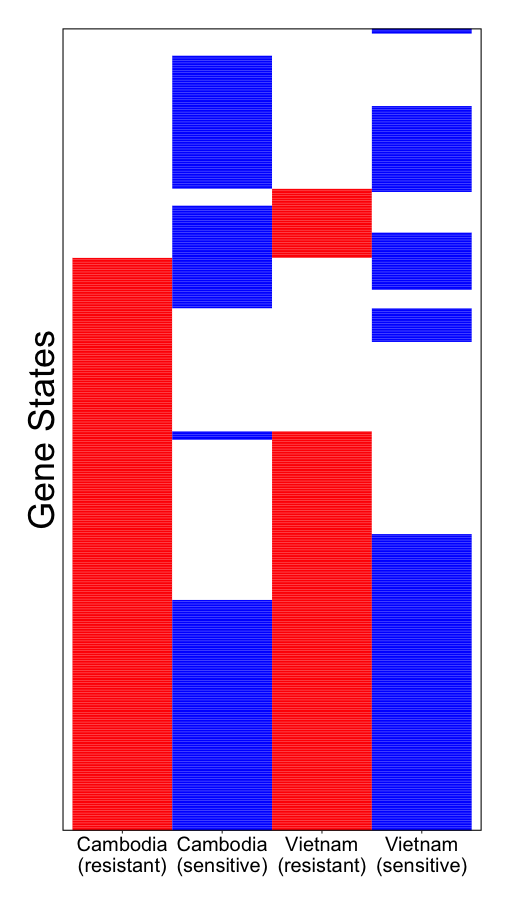

Supplement: Supplementary file 4 — Functional differences in data-driven sensitive and resistant models. Gene states from four condition-specific models, the results of MADE integration, cluster by sensitivity not by location. Active genes in red/blue, with genes removed from expression-constrained models in white. (TIFF 1795 kb) [file 12864_2017_3905_MOESM4_ESM.tiff]

Sensitive  
Resistant

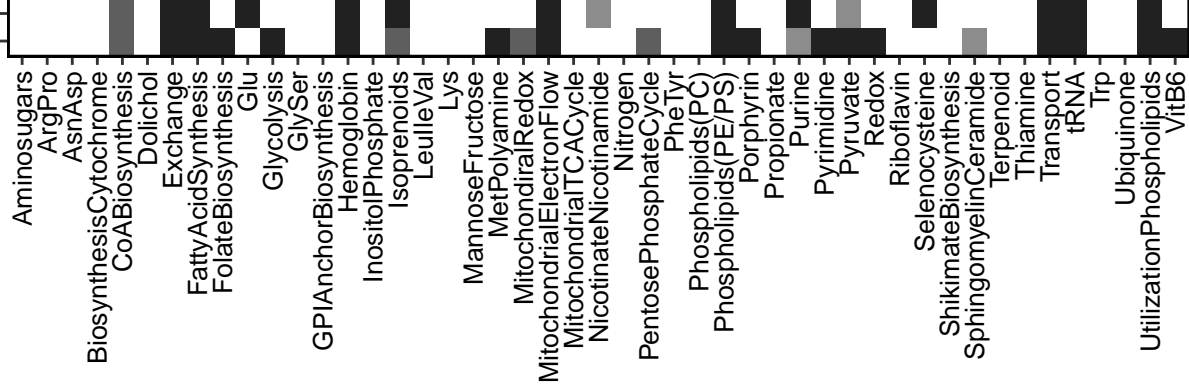

0.001

0.01

0.05

1

Supplement: Supplementary file 5 — Artemisinin sensitive and resistant parasites utilize different metabolic genes and pathways. Enrichment analysis of gene utilization in sensitive and resistant parasite models demonstrates functional differences in expression data integration. Consensus gene utilization from resistant and sensitive models (both Cambodian and Vietnamese datasets) were used and compared to unconstrained model. Black = p < 0.001, grey = p < 0.01, light grey = p < 0.05, white = non significant. Abbreviation key: Aminosugars = amino sugar metabolism; ArgPro = arginine and proline metabolism; AsnAsp = asparagine and aspartate metabolism; BiosynthesisCytochrome = biosynthesis of cytochromes; CoABiosynthesis = coenzyme-A biosynthesis; Dolichol = dolichol metabolism; Exchange = exchange reactions; FattyAcidSynthesis = fatty acid synthesis; FolateBiosynthesis = folate biosynthesis; Glu = glutamate metabolism; Glycolysis = glycolysis; GlySer = glycine and serine metabolism; GPIAnchorBiosynthesis = GPI anchor biosynthesis; Hemoglobin = hemoglobin degradation (including hemozoin formation); InositolPhosphate = inositol phosphate metabolism; Isoprenoids = isoprenoid metabolism; LeuIleVal = leucine, isoleucine, and valine metabolism; Lys = lysine metabolism; MannoseFructose = mannose and fructose metabolism; MetPolyamine = methionine and polyamine metabolism; MitochondrialElectronFlow = mitochondrial electron transport chain; MitochondrialTCACycle = mitochondrial tricarboxylic acid cycle; NicotinateNicotinamide = nicotinate and nicotinamide metabolism; Nitrogen = nitrogen metabolism; PentosePhosphateCycle = pentose phosphate cycle; PheTyr = phenylalanine and tyrosine metabolism; Phosphatidylcholine = phosphatidylcholine metabolism; PhosphatidyletanolaminePhosphatidylserine = phosphatidyletanolamine and phosphatidylserine metabolism; Porphyrin = porphyrin metabolism; Propionate = propionate metabolism; Purine = purine metabolism; Pyrimidine = pyrimidine metabolism; Pyruvate = pyruvate metabolism; [file 12864_2017_3905_MOESM5_ESM.pdf]
